# An assertive, practical, and substantive agenda to catalyse meaningful change

**DOI:** 10.1016/S2214-109X(22)00177-2

**Published:** 2022-04-04

**Authors:** Joseph Kutzin, Suraya Dalil, Helene Barroy, Shannon Barkley, Fahdi Dkhimi, Matthew Jowett, Robert Marten, Inke Mathauer, Bruno Meessen, Susan Sparkes, Ke Xu

**Affiliations:** a World Health Organization, Geneva 1211, Switzerland

The *Lancet Global Health* Commission on financing primary health care^1^ combines a shared vision with practical guidance on how to align health financing with overall reform strategies that place primary care service delivery at the core. The Commission reinforces key messages that WHO has put forward on health financing reforms to enable progress towards universal health coverage (UHC). It then extends these by application to primary care as a critical service delivery element for the progressive realisation of UHC. WHO's guidance on health financing is crystallised into a framework for regular country assessment to inform policy dialogue.^2^ The alignment of the Commission with this guidance is clear, as reflected in the table. While the decision to limit the operational definition of primary health care (PHC) to service delivery platforms was made for the purposes of the Commission, certain key financing issues merit further attention. We point to these towards the end of this Comment.

**Table:** Alignment of the *Lancet Global Health* Commission recommendations with WHO guidance on health financing

**WHO progress matrix desirable attribute**	**Commission recommendation**
Health expenditure is based predominantly on public funding sources	Public resources should provide the core of PHC funding, with minimal reliance on direct payments when services are accessed
Benefit design includes explicit limits on user charges and protects access for vulnerable groups	Public resources should provide the core of PHC funding, with minimal reliance on direct payments when services are accessed
Pooling structure and mechanisms across the health system enhances the potential to redistribute available prepaid funds	Reduce fragmentation, thereby creating an enabling environment for more equitable cross-subsidies between healthy and ill as well as rich and poor, more efficient integration between levels of care, and better coordination with services in (often donor-funded) disease or intervention-specific programmes
Health system and financing functions are integrated or coordinated across schemes and programmes	Reduce fragmentation, thereby creating an enabling environment for more equitable cross-subsidies between healthy and ill as well as rich and poor, more efficient integration between levels of care, and better coordination with services in (often donor-funded) disease or intervention-specific programmes
Resource allocation to providers reflects a combination of population health needs and provider performance	Payment methods should assign resources based on people's health needs and align incentives with people-centred services
Purchasing arrangements are tailored in support of service delivery objectives	Payment methods should assign resources based on people's health needs and align incentives with people-centred services
A set of priority health service benefits within a unified framework is implemented for the entire population	Pooled funds should cover PHC and enable all people to receive it free at the point of use
Health budget formulation and structure support flexible spending and are aligned with sector priorities	Public financial management systems must be flexible and straightforward, enabling managers to respond to the changing needs of patients, families, and communities
Providers can directly receive revenues, flexibly manage them, and report on spending and outputs	Funds flow to and are managed by frontline providers (autonomy)

PHC=primary health care.

From a health financing perspective, perhaps the most far-reaching and potentially influential recommendations in the Commission are (a) to move towards a coherent mixed-provider payment model for PHC with capitation at the core, and (b) to universalise PHC coverage while eliminating or greatly reducing out-of-pocket payments for these services. We agree; here we identify some key issues of policy and implementation alignment that are implied by these directions.

Universality requires that the funding base, particularly but not only in low-income and middle-income countries (LMICs), would rely predominantly on general government budget revenues, regardless of whether they flow directly to providers or via a service purchasing agency such as a health insurance fund. For any payment system paid from government budget revenues to be effective, budget formulation, and execution—the fundamentals of public financial management—will need to function sufficiently well to enable providers to receive a steady, predictable flow of funds^3^ with the ability to manage these flexibly.^4^ This can be facilitated with the design of a programme-based budget that is defined in a way (eg, as access to PHC services) that aligns with the capitation strategy and shifts control and monitoring from inputs to predefined performance indicators that are progressively refined over time.

In reality, a country's starting point for reorienting provider payment to a more coherent set of incentives for “people-centred care” is more complex than the extremes of either line-item budgets or unmanaged fee-for-service, given the fragmentation of revenues flowing from different schemes and programmes that exists in most countries. Thus, a diagnostic of initial payment arrangements^5^ will be essential to create a realistic transition process. As the people-centred payment model of capitation becomes more sophisticated, it should move beyond one level of care and be designed in a way to explicitly encourage provision of services and tasks at lower-level facilities and provider types that are increasingly close to the population they serve. But financial incentives alone are not enough; from early in the reform process, it will be essential to identify service delivery strategies tailored to local needs, including settings challenged by inadequate levels or mix of health workforce, which address underlying performance issues, and then to align the specifics of purchasing with these strategies.

Universalising coverage for PHC services means, in effect, that entitlement to services would have to be entirely or predominantly non-contributory in nature, or done so *de facto* by relying on general revenues to fund coverage for the uninsured in countries relying mainly on social health insurance. This mechanism aligns with a growing consensus^6^, ^7^ about the weakness of contributory-based entitlement (typically in the form of social health insurance) in contexts of limited labour formality.

As noted by the Commission, most out-of-pocket spending in PHC is for medicines. To eliminate or greatly reduce such payments, it is essential that prescribed medicines are made available either without co-payment or with low and explicit limits that are fixed in absolute rather than percentage terms.^8^ This has obvious implications for prioritising generic medicine, management of prescribing patterns, strong price negotiation processes, and where relevant, contracting with private pharmacies.

Universalising PHC combined with the approach of progressing towards a purposively aligned mixed-provider payment system centred on capitation will require, concurrently, taking steps towards a unified or interoperable population (and eventually health service use) database, regardless of affiliation to specific health programme or coverage schemes. This is perhaps a “hidden agenda” for UHC, as unified data systems are a critical input for learning, adaptable (ie, resilient) health systems, reflected in the strategic pathway shown in figure 11 of the report.

Finally, choosing capitation is a political act, because it requires an explicit, up-front decision on the share or amount of the budget that will be allocated to this purpose. Thus, the Commission's recommendations to have applied political economy analysis^9^ as an integral part of policy development is particularly relevant here.

From the perspective of expenditure tracking, interpreting PHC as a service delivery system or platform is appealing because it is measurable. Because PHC is not a category in the System of Health Accounts, estimates have to be constructed based on choices about which expenditure categories to assign to it. A “global” measure of PHC is inherently challenging because countries differ in how they organise PHC, and thus no global measure will be equally relevant to all countries. The global measure used by WHO^10^ was the product of extensive consultation. However, as more countries engage in monitoring PHC spending, and as new service delivery models emerge following the pandemic, ensuring policy relevance and cross-country comparability requires that we periodically revisit the measure; the Commission's recommendations are very helpful for this agenda. As the Commissioners have recognised, what is most important is to improve the quality and increase the frequency with which countries produce their own health accounts. Then, based on how PHC is organised in the country, they can cross-tabulate the “provider” and “function” classifications to assign expenditures to PHC in a way that is most policy-relevant in the national context.

For those of us who work on health financing, considering primary health care only in terms of service delivery is familiar, comfortable ground. The concept of PHC, however, is broader,^11^ and a next generation of work on “financing for health” is needed to reflect this, for example by taking on the challenge of multisectoral budgeting^12^ aimed at addressing cross-cutting public health functions and health determinants emanating from outside as well as inside the health system. As the Commission authors note, we need to adapt our financing levers to population-based essential public health functions across service delivery platforms. In addition, enabling people and communities to take a more proactive role in their health likely requires some rethinking of health financing instruments from a more “demand-side” perspective. The Commission highlights the importance of these issues, but country evidence is limited, and there is great scope for further policy development. Similarly, the design of financing instruments to support health in all policies and an “economy for health”^13^ warrant further attention to lay out an actionable agenda going forward.

There is no global blueprint for how to organise financing to support PHC as the means for the progressive realisation of progress towards UHC. But the fact that we do not know *everything* does not mean that we do not know *anything*. Some ways of doing things are better than others.^14^ The Commission continues and deepens this more assertive approach to health financing. The Commission starts from the position that stronger PHC is the best (more equitable, more efficient) approach for the progressive realization of UHC, and then applies what is known about health financing to provide concrete guidance and clear directionality to governments and international agencies. The Commission's specific emphasis on the political economy of PHC-oriented health financing reforms is important to address the question of why, despite all the years that have passed since 1978, these reforms have not gained traction in so many countries. By clearly stating concrete technical approaches that align to PHC and explicitly recognising the inherently political nature of these processes, the Commission will, we hope, catalyse practical approaches that move beyond rhetoric and agenda-setting to actual implementation.

We declare no competing interests.
